# Advancing Equity for Global Health Author Teams within the Publishing Ecosystem

**DOI:** 10.5334/aogh.5265

**Published:** 2026-09-30

**Authors:** Ella August, Archana Shrestha, Joseph C. Kolars, Pebalo Francis Pebolo, Patricia Garcia

**Affiliations:** 1 Department of Epidemiology, University of Michigan School of Public Health, Ann Arbor, Michigan, USA; 2 Kathmandu University School of Medical Sciences, Kathmandu, Nepal; 3 University of Michigan Medical School, Ann Arbor, Michigan, USA; 4 Department Reproductive Health, Gulu University Faculty of Medicine, Gulu, Uganda; 5 School of Public Health, Cayetano Heredia University, Lima, Peru

**Keywords:** global health, authorship, publishing, equity

## Abstract

The global health literature reflects the need to restructure systems that balance power, remove financial barriers, and ultimately enhance equity, but immediate strategies to enhance equity are often underappreciated, particularly with regard to navigating the publishing ecosystem. Our paper describes how researchers can navigate the publishing ecosystem to create greater equity within their author teams, including those related to communication, supporting junior investigators in building their writing and professional skills, selecting journals that prioritize equity, ensuring transparent recognition of all contributors, and understanding institutional requirements for professional advancement. We also suggest changes in the publishing ecosystem to further enhance equity related to the presentation and recognition of joint authorship, developing a system for identifying predatory journals, and how publishing agreements might better promote equity in authorship. Our advice can help research teams position themselves to achieve greater equity among themselves. Additionally, our suggested changes to the publishing ecosystem will encourage future efforts toward equity.

## Introduction

There is increasing awareness among leaders in global health as well as within global health journals, of the need to better represent authors from low‑ and middle‑income countries (LMICs) [1, 2]. While it is widely understood that the position of an author in the author list conveys varying levels of import and recognition, authors from these regions remain underrepresented in significant authorship positions [3]. This underrepresentation results in missed opportunities to incorporate local perspectives, and researchers in LMICs are thus deprived of professional advancement opportunities [4].

Though the global health literature reflects the need to restructure systems that balance power, remove financial barriers, and ultimately enhance equity [5], immediate strategies to promote equity are often underappreciated, particularly with regard to navigating the publishing ecosystem. The selection of an appropriate journal and the determination of author order must be understood in the context of policies regarding faculty advancement (e.g., promotion, salary increases, etc.) at the author’s academic institutions. In some countries and regions (e.g., China [6]), authorship position is linked to financial compensation provided by the authors’ affiliated institutions. These considerations are especially important for those new to global health or early in their careers. Further, much of the literature on practices to enhance authorship equity is fragmented, as it targets different readerships including authors, journal editors, librarians, academic institutions, and standard‑setting organizations like the International Committee of Medical Journal Editors (ICMJE).

This paper addresses these gaps. Rather than focusing solely on how author teams can promote equity when determining position on a paper, we situate our advice within the broader publishing ecosystem, equipping early‑stage researchers with a more informed understanding of the affordances and constraints posed by journals, publishers, academic and research institutions, and organizations like ICMJE that shape publishing guidelines and standards. Additionally, we incorporate advice about generative artificial intelligence (AI), which is largely absent from the existing literature on this topic. Finally, we provide suggestions to help the publishing ecosystem encourage efforts toward greater equity.

## Strategies to Promote More Equitable Authorship

We propose several approaches to fostering more equitable authorship practices involving collaborations of researchers from LMICs and high‑income countries (HICs). These approaches include engaging in explicit communication around power imbalances and authorship roles, supporting junior investigators in building their writing and professional skills, selecting journals that prioritize equity, ensuring transparent recognition of all contributors, and understanding institutional requirements for professional advancement. (See Box for a summary of these strategies.)

**Box. Summary of Author Team Strategies to Enhance Equity in Authorship**

- Authorship planning
  - Discuss opportunities for authorship early in the research process
  - Provide clear criteria for authorship and clarity on how authorship roles and order will be determined within the research team
  - Consider the most appropriate authorship criteria in terms of inclusiveness
  - Consider joint authorship as a way to reflect collaborative contributions
  - Explore the creation of a collaborative or study group if relevant
  - Investigate and understand how contributorship is reported
- Equity and inclusion
  - Acknowledge power dynamics between authors from HICs and LMICs
  - Assist all authors in understanding the implications of authorship position to their institutions and for their careers
  - Use the acknowledgments section to recognize contributions from non‑author collaborators
  - Set a goal for all team members to benefit from scholarly publishing activities
  - Use reflexivity statements to reflect on how colleagues from LMICs can be supported for greater participation as authors
- Support for early‑career investigators
  - Engage in active listening with team members to understand their perspectives and needs
  - Encourage early‑career investigators to understand their institution’s requirements for professional advancement and share these with the team to support their success
  - Provide protected time for writing and receiving feedback on manuscript drafts
- Resources
  - Take advantage of free resources such as the Rising Scholars^1^ (https://risingscholars.net/en/) platform
  - Partner with external pre‑publication support when appropriate
  - Train second‑language speakers to use generative AI for copyediting assistance
- Journal selection
  - Avoid predatory journals
  - Be aware of article processing charges and how they might impact the ability to publish
  - Choose a journal that aligns with your team’s needs, considering all the above aspects

Research teams should discuss opportunities for authorship participation early in the project, even during grant development. All team members should be made aware of the criteria for authorship, and how authorship roles and order will be determined. Frassl et al. [7] provide valuable guidance for planning these discussions, which may need to be revisited as circumstances evolve. Inherent power dynamics between individuals from HICs and those from LMICs must be understood and acknowledged up front. Authors from LMICs often do not feel they are on equal footing despite the intentions of collaborators from HICs [8]. Setting a goal for all study team members to benefit from scholarly publishing activities and engaging in active listening can help make these conversations more productive [7, 8].

Investigators and institutions from HICs may need to support their colleagues from LMICs in developing the skills necessary to fully participate as authors. Reflexivity statements typically require researchers to disclose how the study questions and design were developed, describe how those from HICs supported local capacity, and explain how authorship was determined—with particular emphasis on gender balance, inclusion of early‑career researchers, and recognition of local leadership [9]. Such training can be provided through mentorship by senior investigators, free resources like the Rising Scholars platform, or external pre‑publication support (e.g., PREPSS.org). Additionally, generative AI tools can promote equity; for instance, free platforms like ChatGPT can be used for copyediting [10]. Institutions can also establish policies on authorship, including mechanisms for conflict resolution to address issues related to power imbalance and bias.

Authors should be cautioned to avoid predatory journals, defined as “entities that prioritize self‑interest at the expense of scholarship and are characterized by false or misleading information” [11]. Such journals do not adhere to best editorial and publication practices, lack transparency, and often employ aggressive and indiscriminate solicitation practices. They pose barriers to legitimate publication by luring researchers into submitting their work under false pretenses. Many publish articles by individuals who may or may not have an academic degree and are paid hourly or monthly to churn out papers.

Journals differ in their authorship policies, and the team can choose one that maximizes opportunities for equity. Some journals define authorship more inclusively; for example, while many journals adhere to the ICMJE criteria, others like PLOS Global Public Health use the more inclusive criteria developed by McNutt et al. [12].

Journals can also be selected based on whether they allow for joint author positions, such as co‑first, co‑last, and co‑corresponding authors. These joint authorship arrangements maximize opportunities to meaningfully participate and receive recognition, ideally pairing one team member from an HIC with another from the LMIC team. One productive way to pair co‑first authors is by including a student from an HIC alongside an author from an LMIC, maximizing learning, experience, and credit for both parties.

Another journal policy to consider is the ability to accommodate large author lists. Including 30 or more authors may be allowable, providing more inclusivity and credit. Establishing a collaborative or study group is another way to include a large number of authors. While being part of a collaborative author group may not be ideal for researchers or faculty members who need visibility to advance their careers, it can be an effective way to offer recognition to field workers or others who deserve credit, but whose careers do not depend on publications.

A final journal policy to review is the article processing charge. Some journals offer partial fee waivers for authors from LMICs, while requiring authors from HICs to cover a portion of the charge.

Transparent reporting of each author’s contributions—such as conceptual development, data analysis, drafting, and editing—through contributorship statements like CRediT [13] provides an effective means of recognizing each author’s efforts. In some cases, individuals may have assisted with the research without meeting the criteria for authorship. Generously recognizing such individuals in the acknowledgments section is an equitable and inclusive practice [14].

Junior investigators should be encouraged to understand their institution’s requirements for professional advancement. For example, advancing in rank may require a certain number of first‑authored papers or other criteria. Sharing these standards with the team can help in collectively striving toward these goals. Providing protected time for writing and receiving feedback from mentors can be enormously beneficial in supporting less experienced authors who are overburdened with time commitments. These arrangements can be negotiated with the research team and affiliated institutions.

Finally, we caution author teams against authorship tokenism, the inclusion of authors in name only, as it can be as harmful to junior investigators as exclusion. Following the advice in our paper around transparency about process and author contributions, understanding authorship requirements, supporting junior authors in building their writing and professional skills, and promoting agency and engagement collectively works to redistribute the power within author teams, which in turn reduces the likelihood of tokenism. That said, if our advice is not implemented thoughtfully, it could inadvertently result in authorship tokenism without meaningful contributions.

## Future Directions

We recommend several changes to improve equity in authorship practices (see Box 1 for summary). With regard to the broader publishing ecosystem, we also recommend (1) standardizing how joint authorship is represented across journals, other document types, and digital platforms; (2) developing an effective system for identifying predatory journals; (3) ensuring that institutions fairly recognize joint authorship in their evaluations and reward structures; and (4) negotiating publishing agreements that promote equity in authorship.

While joint author positions such as co‑first provide an excellent opportunity to share credit, standardized practices for denoting such arrangements would further enhance their visibility and impact. Currently, practices vary widely across article headers, citation lists, and academic databases. For example, some journals (like PLOS One^2^) display co‑first authorship in the article header, and others (e.g., Nature^3^) use different designations. In citation lists, joint authors are typically not flagged. In academic literature databases, recognition of joint authorship positions is similarly inconsistent [15]. This inconsistency means that shared contributions are frequently invisible or ambiguous depending on where and how a paper is accessed.

As Dubansky and Omary [16] have argued, explicit and consistent acknowledgment of joint authorship is overdue. Standardizing the way joint authorship positions are identified would ensure that shared prominent authorship positions are recognized regardless of the platform or context in which a publication is encountered—which has particular implications for early‑career researchers whose contributions might otherwise go unrecognized. For example, the Journal of Hepatology bolds the names of authors sharing joint first or last author positions in reference lists [16]. Applying a uniform and explicit label (e.g., “co‑first authors”) across publications in headers and in reference lists of published articles would improve recognition and visibility. We further recommend standardized practices for representing joint authors in curricula vitae and faculty web pages. Finally, a system to identify joint authorship in library search engine and PubMed results should be established [15].

There is a clear need for developing a more effective system to identify predatory journals. A recent article by Siebert et al. presented an ambitious proposal that would have ICMJE establish a certification system to monitor publishing standards [17]. Regardless of whether the ICMJE is able or willing to undertake this task, we need a monitoring system—a comprehensive list of either predatory journals or non‑predatory journals which is updated regularly to guide researchers.

Recognition for promotion and pay raises should be generously granted in cases of joint authorship. For instance, co‑first authorship should be considered equivalent to sole first authorship for researchers and faculty members.

Importantly, evidence demonstrating that the proposed approaches would achieve greater recognition and equity is needed. However, we acknowledge the complexity of quantitatively demonstrating the impact of such approaches as Faure et al. point out in their scoping review on international health collaborations [18].

Finally, open‑access publishing agreements negotiated by academic institutions play a significant role in balancing power between publishers and affiliated researchers. However, many such agreements are negotiated by institutions in HICs. The impact of such agreements on authorship should be considered through the lens of equity. For example, the requirement for an author affiliated with a participating HIC institution to be the corresponding author may encourage an HIC author to fill this role, potentially excluding authors from LMICs. Academic and research institutions should negotiate these agreements to allow for flexibility in author roles.

## Conclusion

Publishing in the global health space can present entrenched barriers to equity; however, meaningful opportunities to advance equity within the publishing ecosystem exist. Implementing key changes in this realm can further promote equitable practices and outcomes.

## Footnotes

1 https://risingscholars.net/en/.

2 For example, https://journals.plos.org/plosone/article?id=10.1371/journal.pone.0000396.

3 For example, https://www.nature.com/articles/s41586-020-2180-5.
